# Gene therapy in glaucoma-part 2: Genetic etiology and gene mapping

**DOI:** 10.4103/0974-620X.64227

**Published:** 2010

**Authors:** Mohamed Abdel-Monem Soliman Mahdy

**Affiliations:** Department of Ophthalmology, Rusatq Hospital, Sultanate of Oman, and Al-Hussein University Hospital, Faculty of Medicine, Al-Azhar University, Cairo, Egypt

**Keywords:** Gene therapy, genetic diagnosis of glaucoma, glaucoma

## Abstract

Glaucoma diagnosis, identification of people at risk, initiation of treatment and timing of surgical intervention remains a problem. Despite new and improving diagnostic and therapeutic options for glaucoma, blindness from glaucoma is increasing and glaucoma remains a major public health problem. The role of heredity in ocular disease is attracting greater attention as the knowledge and recent advances of Human Genome Project and the HapMap Project have made genetic analysis of many human disorders possible.

Glaucoma offers a variety of potential targets for gene therapy. All risk factors for glaucoma and their underlying causes are potentially susceptible to modulation by gene transfer.

The discovery of genes responsible for glaucoma has led to the development of new methods of Deoxyribonucleic acid (DNA)-based diagnosis and treatment. As genetic defects responsible for glaucoma are identified and the biochemical mechanisms underlying the disease are recognized, new methods of therapy can be developed. It is of utmost importance for the ophthalmologists and glaucoma specialists to be familiar with and understand the basic molecular mechanisms, genes responsible for glaucoma and the ways of genetic treatment.

**Method of Literature Search:**

The literature was searched on the Medline database, using the PubMed interface.

## Article Outline

- **Genetic Etiology of Glaucoma**
- **Genes Associated with Forms of Glaucoma with Mendelian Inheritance**

A. **Childhood Glaucoma**
  - Congenital Glaucoma
  - Anterior Segment Dysgenesis Syndromes
    1. Axenfeld-Rieger,
    2. Nail-patella Syndrome,
    3. Aniridia and PAX6 Gene
B. **Primary Open – Angle Glaucoma (POAG)**
  - Genetic Loci for POAG
    1. GLC1A (TIGR/Myocilin)
    2. GLC1B
    3. GLC1C
    4. GLC1D
    5. GLC1E
    6. GLC1F
    7. GLC1G
    8. GLC1J, GLC1K and other loci
  - Candidate Genes Causative of POAG
  - Genes Associated with POAG and Gene-gene Interaction
C. **Pigment Dispersion Syndrome**
D. **Pseudoexfoliation Syndrome and Glaucoma**
  - Conclusion; Developing a Diagnostic Panel for Patients at Risk for Glaucoma

## Introduction

Glaucoma is the 2^nd^ most common cause of blindness in the world as determined by the world health organization (WHO). In Oman the rate of glaucoma cases reported by ophthalmologists was 1.14/1000. 11.5% of estimated blindness in Oman was due to glaucoma.[1,2]

The role of heredity in ocular disease is attracting greater attention as the knowledge and recent advances of Human Genome Project and the HapMap Project made genetic analysis of many human disorders possible (the Human Genome was completed in April 2003 and the International HapMap Project in 2005).[3–5]

The discovery of genes responsible for glaucoma is opening the possibility of new methods of DNA-based diagnosis and treatment. As genetic defects and biochemical mechanisms responsible for glaucoma are, new methods of therapy can be developed. In some cases, understanding the responsible biochemical abnormalities may indicate metabolites, either small molecules or enzymes that could be administered to correct the problem. Using new technologies, novel methods of diagnosis and treatment, based on the genetic defects responsible for glaucoma, will allow individuals at risk for the disease to be identified and successfully treated before irreversible damage to the nerve has occurred.[6]

As large numbers of patients with genetic ocular disease especially glaucoma are identified, public demands for prevention, treatment and counseling increase. Consequently ophthalmologists, like other medical specialists are expected to be familiar with the clinical manifestations, transmission patterns, diagnostic techniques, and therapeutic modalities of heritable disorders in their specialty.[7] In a previous article in the Oman Journal of ophthalmology, the basic mechanisms and molecular genetic had been described. In this context this article is written to further highlight the overview of gene therapy in glaucoma especially the genetic etiology and mapping in various type of glaucoma.[8]

## Genetic Etiology of Glaucoma

Inheritance of glaucoma could be Mendelian autosomal-dominant (AD), autosomal recessive (AR) trait, or as a complex multifactorial trait. Early onset forms of the glaucoma usually inherited as AR trait, while adult onset forms of the disease exhibit a heritable susceptibility consistent with a complex multifactorial trait inheritance. Recent advances in genetic approaches had helped to define the underlying molecular events responsible for some mendelian forms of the disease and had identified the chromosome locations of genes that are likely to contribute to common complex forms.[9]

Genes associated with different forms of glaucoma can be detected in the human genome using both large affected pedigrees and standard linkage analysis.[9] The simplicity of genetic approaches had lead to substantial success and most of the genes currently known to be associated with various forms of glaucoma were identified using these methods [Table 1].

**Table 1:** Clinical condition, and chromosomal locations of genes associated with glaucoma[49]

*Clinical condition*	*Locus (Gene)*	*Chromosome location*	*Inheritance pattern*
Early-onset POAG	GLC1J	9q22	AD
	GLC1K	20p12	AD
Adult-onset POAG	GLC1B	2cen-2q13	AD
	GLC1C	3q21-24	AD
	GLC1G (WDR36)	5q22	AD; complex
	GLC1D	8q23	AD
	Locus pending	14q11	Complex
	GLC1I	15q11-q13	Complex
	GLC1F	7q35	AD
Early-and adult-onset POAG	GLC1A (MYOC)	1q23	Early-onset; AD Adultonset; complex
Adult-onset POAG; low-tension glaucoma	GLC1E (OPTN)	10p15-p14	AD
Pigment dispersion syndrome	GPDS1	7q35-q36	AD
Congenital glaucoma	GLC3B	1p36	AR
	GLC3A (CYP1B1)	2p21	AR
Nanophthalmos	NNO1	11p	AD
	VMD2	11q12	AD
	MFRP	11q23	AR
Rieger syndrome	RIEG1 (PITX2)	4q25	AD
	RIEG2	13q14	AD
Iridodysgenesis	IRID1 (FOXC1)	6p25	AD
Aniridia	AN2 (PAX6)	11p13	AD
Glaucoma associated with nail-patella syndrome	(LMX1B)	9q34	AD

AD - Autosomal dominant; AR - Autosomal recessive; POAG - Primary openangle glaucoma

## Relationship Between Different Clinical Forms of Glaucoma and Genetic Mendelian Inheritance

Typically, early-onset forms of glaucoma are inherited as mendelian dominant or mendelian-recessive traits. These include early-onset open angle glaucoma;[10–12] congenital glaucomas[13] developmental glaucomas including Rieger syndrome,[14,15] glaucoma associated with nail patella syndrome,[16] and nanophthalmos,[17] and glaucoma associated with pigment dispersion syndrome.[18,19] Adult onset forms of the disease usually inherited as a complex multifactorial trait.

## Childhood Glaucoma

### 

#### Congenital glaucomas

Primary congenital or infantile glaucoma (GLC3): Usually occurs in early childhood, within the first year of life, but may develop later up to 3 years of age. It is usually inherited as an autosomal recessive trait and is prevalent in countries where consanguinity is common.[20,21] In patients with congenital glaucoma, the development of the anterior segment of the eye and aqueous humor outflow pathways is abnormal, causing high IOP. Using consanguineous pedigrees from Saudi Arabia and Turkey, defects in the cytochrome P4501B1 (*CYP1B1*) gene coding for a protein that is a member of the cytochrome P450 family were found in individuals affected with congenital glaucoma. Subsequently, mutations in this gene have also been found in patients from many other countries including those with more heterogeneous populations, such as the United States and Brazil. Sarfarazi *et al*.[22] mapped a locus for primary congenital glaucoma, GLC3A, to 2p21. Mutations in the gene for cytochrome P4501B1 (*CYP1B1*) were identified, and account for most cases of autosomal recessive congenital glaucoma, CYP1B1 could be the predominant cause of primary congenital glaucoma (PCG) in the Omani population (78%).[22,23] Numerous cytogenetic reports indicate other chromosome regions that may harbor congenital glaucoma genes, and autosomal dominant forms of congenital glaucoma have been identified.[24] Mapping studies in eight families with congenital glaucoma have revealed a second locus, GLC3B, at 1p36.[25]

The cytochrome P450 (a product of the *CYP1B1*gene) participates in the metabolism of many compounds, including 17B-estradiol. Loss of protein function is probably the underlying genetic mechanism, as most of the mutations are deletions, insertions, or missense mutations occurring in highly conserved protein regions that are necessary for its function.[20,26] It has been hypothesized that alterations in the metabolism of estrogens may be the basis for the ocular abnormalities associated with defects in this gene.[27] Most patients with congenital glaucoma caused by mutations in *CYP1B1* have severe form of the disease.[21] Also, a cytochrome P450-dependent arachidonic acid metabolite inhibits Na+, K+-ATPase in the cornea and may regulate corneal transparency and aqueous humor production.[28]

#### Anterior segment dysgenesis syndromes

Linkage studies suggest that genes responsible for anterior segment developmental abnormalities are located on chromosomes 13q14,[15] 4p,[29] 16q,[30] and 20p. In mice, several genes have been suggested as responsible for ocular developmental defects leading to glaucoma, including *Bmp4, Foxe3*, and *Tgfb2*[31] [Table 2].

**Table 2:** Anterior segment dysgenesis and glaucoma and associated genes[81]

*Human disease*	*Chromosome location*	*Gene*
Anterior segment dysgenesis, umbilical, and teeth abnormalities	4q27	PITX2
Anterior segment dysgenesis, lens and corneal opacities	10q24-25	PITX3
Anterior segment dysgenesis, teeth abnormalities, cardiac abnormalities	6p25	FOXC1
Anterior segment dysgenesis, lens abnormalities	1p33	FOXE3
Anterior segment abnormalites, Nail-patella syndrome, glomerular nephropathy	9q23	LMX1B
Aniridia	11p13	PAX6

Axenfeld-Rieger’s syndrome: These forms of congenital glaucoma are associated with abnormal development of the anterior segment of the eye. Axenfeld-Rieger syndrome, is characterized by malformations of the anterior segment as well as systemic involvement (facial and dental abnormalities). When the systemic features are lacking, it is termed Rieger (or Axenfeld-Rieger) anomaly. Although Axenfeld-Rieger anomaly and Rieger syndrome share similar ocular features, they are distinct entities on the genetic level.

Axenfeld-Rieger anomaly, mapped to 6p25, is associated with mutations in the FKHL7 gene that codes a transcription factor containing a fork-head domain, the gene responsible is *FOXC1*.[14,32] Initial localization of the *RIEGl* gene, responsible for Rieger syndrome, was aided by cases of *4q-deletion* that also demonstrated the Rieger’s phenotype. Then it was further narrowed the locus to the 4q27 region which contain the gene *PITX2. RIEGl* was found to be a homeobox gene (*PITX2* gene), namely a gene containing a sequence of about 180 DNA base pairs that are highly conserved throughout evolution.[33] These genes are thought to play an important role in development. A second locus involved in the Rieger phenotype was mapped to 13q14.[15] It is interesting to note that a gene for iridogoniodysgenesis, distinct from FKHL7, but in close proximity to it was identified at the 6p25 locus.[32]

Defects in the *FOXC1* gene are found in patients with anterior segment dysgenesis.[32,34] Patients with defects in both of the *PITX2* and *FOXC1* genes may also have associated systemic defects involving the teeth, facial bones, heart, and umbilicus.

**Nail-patella syndrome** is a systemic developmental disease associated with glaucoma caused by defects in *LMX1B*.[35] An autosomal-dominant form of nanophthalmos associated with vitroretinochoroidopathy has been shown to be caused by abnormalities in the *VMD2* gene.[36] The genes responsible for these disorders participate in the regulation of gene expression during development,[37,38] specifically in the development of the periocular mesenchyme, which includes neural crest– and cranial paraxial mesoderm–derived cells.[38] The DNA defects lead to loss of function of the protein and haploinsufficiency.[33,34] Intrafamilial variability and variability in phenotypic expressivity may be caused by dosage effects or by the coexistence of other genes that can modify the expression of the trait.

**Aniridia, nanophthalmos and PAX6 gene:** Recent work suggests that *PAX6* gene is a master control gene for eye formation throughout the animal kingdom, regulating the expression of other genes in time and space during embrygenesis.[39] Abnormalities in the *PAX6* gene cause aniridia, as well as a spectrum of iris abnormalities related to glaucoma.[40] This gene, situated at 11p13, has been implicated in a heterogeneous group of anterior segment anomalies, including Peter’s anomaly, autosomal dominant keratitis, congenital cataract with late onset corneal dystrophy, isolated foveal hypoplasia and aniridia.[41]

## Primary Open-angle Glaucoma

Primary open-angle glaucoma (POAG) is the most common form of glaucoma, accounting for approximately 3% of visual impairment in white and 7.9% of African Americans.[42,43] According to age of onset, POAG is divided into juvenile onset POAG (JOAG) and adult-onset POAG, with overlapping clinical presentations. JOAG, which develops before the age of 35, is a rare disorder that usually requires surgical therapy.[44–46] JOAG is typically inherited as an autosomal dominant trait, whereas adult-onset POAG is inherited as a complex trait.[45]

### 

#### Genetic loci for primary open angle glaucoma

To date, at least 20 genetic loci for primary open angle glaucoma (POAG) have been reported [Table 3].[47,48] Among them, 11 chromosomal loci had been designated GLC1A to GLC1K by the HUGO Genome Nomenclature Committee (www.gene.ucl.ac.uk/nomenclature). Only 3 of them (GLC1A, GLC1J and GLC1K) contributed to JOAG, while the others contributed only to adult-onset POAG.[11,49] The first gene identified as playing a role in Juvenile-onset POAG at the *GLC1A* locus mapped at chromosomal region 1q21-31.[11] Stone and colleagues identified mutations in a candidate gene that mapped to this region, in patients with the POAG.[50] The protein encoded by this gene had been independently characterized as “trabecular meshwork-induced glucocorticoid response protein” (TIGR) in trabecular meshwork cells in culture.[51] The same protein, named Myocilin, had also been localized to the outer segment cilium of photoreceptors by Kubota and colleagues.[52]

**Table 3:** Primary open angle glaucoma genes, loci and chromosomal locations[93]

*Gene identified*	*Locus name*	*Chromosomal location*
MYOC	GLC1A	1q21−q31
OPTN	GLC1E	10p15−p14
WDR36	GLC1G	5q22.1
−	GLC1B	2cen−q13
−	GLC1C	3q21−q24
−	GLC1D	8q23
−	GLC1F	7q35−q36
−	GLC1H	2p16.3−p15
−	GLC1I	15q11−q13
−	GLC1J	9q22
−	GLC1K	20p12
−	−	2p14
−	−	2q33−q34
−	−	3p21−p22
−	−	10p12−p13
−	−	14q11
−	−	14q21−q22
−	−	17p13
−	−	17q25
−	−	19q12−q14

Only 3 causative genes are identified from these loci: Myocilin (*MYOC*), Optineurin (*OPTN*) and *WD* repeat domain 36 (*WDR36*), which together account for less than 10% of POAG. Only a portion of POAG follows Mendelian inheritance, and a considerable fraction results from a large number of variants in several genes, each contributing small effects.[53] The genetics of POAG are therefore complex. Both genetic and environmental factors are implicated in its etiology. For the 3 known POAG genes, only *Myocilin* (*MYOC*) is established as directly glaucoma causative, while the roles of *Optineurin* (*OPTN*) and *WD repeat domain 36* (*WDR36*) are still unclear due to conflicting evidence. *MYOC* mutations account for 1.1%–4% of POAG, depending on the population. Up to 20% of patients with early-onset POAG and 3%-5% of patients with adult-onset POAG have defects in this gene.[54,55] Some mutations are specifically associated with early onset disease, while others are more common in adult-onset patients. One study has suggested that heterozygous defects of the *CYP1B1* gene can influence the severity of disease caused by mutations in *MYOC*.[56]

Single mutation in the TIGR gene may cause a variety of phenotypes from ocular hypertension (OHT) to (JOAG) to chronic open angle glaucoma (COAG). The variable expressivity in the JOAG and COAG patients may simply be a result of the age at which these candidates are affected.[57] It is likely that multiple genes (independently or in combination) are responsible for the heritability of POAG. The variability in the age of onset of the disease, the apparent incomplete penetrance and the prevalence of the disease all suggest that more than one gene may be responsible for the disorder and POAG is not inherited as a simple single gene disorder but as a complicated “complex trait”.

#### GLC1A (TIGR/Myocilin)

GLC1A is the 1^st^ locus linked to POAG phenotype. Most GLClA linked families have been characterized by a severe and aggressive form of open-angle glaucoma with an early onset, usually before age 40, peak IOPs greater than 30 mm Hg, and severe damage to the optic nerve. Many individuals required filtering procedure.[58,59] It is inherited in an autosomal dominant fashion. The GLC1A region was originally mapped to chromosome 1q21-31.[11] *Myocilin* gene is identified as the gene mutated in this disorder.[50]

#### GLC1B

This is the second locus found to be linked to the POAG phenotype.[60] It is mapped to the centromeric portion of chromosome 2 (2 cen- q13). In contrast with GLClA families and individuals, those with the GLClB locus appear to be associated with a milder phenotype of adult onset POAG, lower peak-IOP, and older age of onset. Fifty percent of GLClB-affected individuals never have had a measured IOP higher than 22 mmHg whereas most of the remaining showed maximal elevations in the range of 22 to 30 mm Hg. The onset was usually in the late forties, with-a good response to medical therapy. This phenotype raises the question of whether normal tension glaucoma (NTG) is associated with this locus. If this is the case, the GLClB gene, once identified, may shed light on pathophysiologic mechanisms underlying the susceptibility of some optic nerves to damage at normal IOPs. No gene has yet been identified for this condition.

#### GLC1C

The GLC1C is mapped within to chromosome 3 within a locus chromosome 3q21–q24. Affected family members in the single pedigree shown to harbor the GLClC gene have glaucoma with high pressures, late onset, visual field loss and/or a cup disc ratio greater than normal and a moderate response to medication.[61] The average age of onset is over 40 years therefore classifying GLC1C as adult onset glaucoma. Although, apparently a rare mutation, the phenotype presented by GLClC patients is more typical of POAG as opposed to the younger onset of GLClA and the low IOPs of GLClB. Thus, it is possible that the GLClC gene will provide insight into the pathophysiologic mechanism of the more typical high IOP, late-onset, POAG.

#### GLC1D

The GLC1D locus was mapped to 8q23, this locus was identified through a linkage study of four generation North American family with adult-onset POAG.[62] The phenotype in this family appeared to be variable, with onset of visual field loss in middle age, followed by modest elevation of IOP and progression of the disease in older individuals. Little is known about the GLClD locus. To date, only a single family is known to harbor this 8q23 mutation.[61]

#### GLC1E

The GLC1E locus was mapped to 10p15–p14, this was identified in a large British family in which 15 of 46 family members were affected with NTG.[63] The age at diagnosis in this family varied between 23 and 65 years. The IOPs in the patients varied but were generally in the normal range with high cup disk ratios, visual field loss and changes of the optic nerve head. The candidate gene was recently identified as *Optineurin* (*OPTN*) based on its physical location in this region and its expression in retina.[64] It was the *second gene identified for POAG*.

#### GLC1F

Genome-wide scan of a large adult-onset POAG family spanning four generations mapped the GLC1F locus to 7q35–q36.[65] All 10 living patients in the family had POAG with grade IV gonioscopy, IOPs of 22 mm Hg or more, and vertical cup-disc ratios of 0.6 or more. Five of them had abnormal visual fields. Interestingly, a gene for pigment dispersion syndrome (PDS) was recently shown to occupy the same 7q35-36 locus.[19] One may only speculate on whether the same gene or different close-by genes are involved in these seemingly very different phenotypes.

#### GLC1G

In a genome-wide scan study, this new adult-onset POAG locus was mapped to 5q33–q35. The candidate gene for GLC1G was identified as *WDR36*.[66] It was *the third POAG gene*. Intriguingly, no coding variations of the *WDR36* gene were found in 2 of the 7 GLC1G linked families, indicating a possibility of non-coding variations or of another POAG gene at GLC1G.[66] New data resulting from discrepancies between the genetic and physical maps positioned the upper boundary for this locus to 5q21.3.[66,67] Although the function of the protein Product of this gene is unknown and the role of this protein in glaucoma remains to be confirmed,[68] prior studies suggest that it may participate in immune responses. Other studies had also suggested that glaucoma may be influenced by immune reactivity.[69] Interestingly, recent evidence suggests that mutations in the *WDR36* gene are not an independent cause of glaucoma but may modify the severity of the disease in an affected person.[70]

#### GLC1J and GLC1K and other loci

Two JOAG loci were mapped to 9q22 and 20p12.[49] The locus on 9q22 was designated GLC1J. The 20p12 locus was designated GLC1K. Of the 15 families sufficient for haplotype analysis, haplotypes were consistent with GLC1J in 7 families, with GLC1K in 5 families, and with linkage to both loci in 3 families.[53]

Recent research mapped a novel JOAG locus to 5q22.1–q32 in a large autosomal dominant JOAG family from the Philippines. This 5-generations family had a total of 95 members, in which 22 were affected with JOAG. Complete ophthalmic examination was given to 27 family members, in which 9 were confirmed JOAG patients.[71] Also, a genome wide scan of 218 affected sibling pairs by using IOP as a continuous trait in linkage analysis identified two potential loci for IOP.[72]

#### Candidate genes causing POAG

In Caucasians about 2%–4% of POAG cases are due to MYOC mutations,[73] although it can be as high as 36% in JOAG families.[74] Mutations in OPTN, arguably the second POAG gene, were initially found in 16.7% of families with hereditary and adult onset POAG, and 12% of sporadic patients with POAG. The majority of them had IOP of less than 22 mm Hg.[64] Two subsequent studies on Caucasian POAG patients, one study involving 801 patients of variable age onsets[75] and one involving 86 adult-onset patients,[76] reported no glaucoma causing mutations in *OPTN*. In 148 Japanese patients with NTG and 165 with HTG, again no specific glaucoma-causing mutations in *OPTN* were identified.[77] The third gene for POAG was characterized as *WDR36* at GLC1G.[66] In the original study, four mutations were found to be associated with more than 5% of all sporadic cases of POAG.[66]

#### Genes associated with POAG and gene-gene interaction

At least 16 POAG-associated genes have been reported from association studies [Table 4].[78,79] A couple of genes have been investigated in multiple association studies. However, conflicting findings were reported in different studies. The role of these genes in the etiology of POAG is still controversial. It is not clear yet whether the inheritance pattern of POAG is monogenic or complex polygenic. The following genes are an example of such genes;

**Table 4:** Associated genes for POAG[93]

*Gene name*	*Gene symbol*	*Chromosomal location*
Angiotensin II receptor, type 2	AGTR2	Xq22−q23
Apolipoprotein E (107741)	APOE	19q13.2
Cyclin-dependent kinase inhibitor 1A	CDKN1A	6p21.2
Cytochrome P450, subfamily I, polypeptide 1	CYP1B1	2p22−p21
Endothelin receptor, type A	EDNRA	4q31.2
Glutathione S-transferase, mu-1	GSTM1	1p13.3
Insulin-like growth factor II	IGF2	11p15.5
Interleukin 1-beta	IL1B	2q14
5,10- methylenetetrahydrofolate reductase	MTHFR	1p36.3
Nitric oxide synthase 3	NOS3	7q36
Natriuretic peptide precursor A	NPPA	1p36.2
Oculomedin	OCLM	1q31.1
Optic atrophy 1	OPA1	3q28−q29
Transporter, ATP- binding cassette, major histocompatibility complex, 1	TAP1	6p21.3
Tumor necrosis factor	TNF	6p21.3
Tumor protein p53	TP53	17p13.1

**Apolipoprotein E (*APOE*)** had been reported to be a potent modifier gene for POAG.[79] The promoter polymorphism –219T>G was associated with increased optic nerve damage, while –491A>T interacted with a MYOC polymorphism, –1000C>G (MYOC.mt1), to increase IOP in POAG patients. The APOE ε4 allele increased risk of NTG.[80]

**Optic atrophy 1 (*OPA1*)** was reported to associate with NTG in the British population. Two single nucleotide polymorphisms (SNPs) in the intron 8 of OPA1, IVS8 + 4C>T and IVS8 + 32T>C, were strongly associated with the occurrence of NTG but not with HTG.[81]

**Cytochrome P4501B1 (*CYP1B1*)** had been considered as a modifier gene for *MYOC* expression in JOAG patients.[82] Carriers with both mutations had early disease onset. Another study in a French population demonstrated *CYP1B1* mutations were more prevalent in JOAG patients than controls.[83,84]

**Tumor protein p53 (*TP53*)** was originally reported to be associated with POAG in a Chinese population.[85] The cytosine residue at codon 72 of the *TP53 gene* was significantly more common in POAG patients than controls.

**Tumor necrosis factor (*TNF*)** was associated with POAG. The promoter polymorphism –308G>A was significantly higher in POAG patients than in controls. A possible interaction between polymorphisms in the *OPTN* and *TNF* genes was identified to increase the POAG risk.[86] carriers with minor alleles of *TNF*/–863C>A, and *OPTN*/ c.603T>A were more common among POAG patients. These interactions between *TNF* and *OPTN* were found to worsen visual fields in POAG patients.

There is increasing evidences that the possible interaction between *MYOC*, *OPTN* and *APOE* might contribute to the development of POAG, indicating a polygenic etiology.[87]

## Pigment Dispersion Syndrome

Approximately 50% of the individuals with clinical evidence of pigment dispersion syndrome will develop glaucoma. In humans the disease can be sporadic or inherited, with most pedigrees demonstrating autosomal-dominant inheritance patterns.[18,19] In pigment dispersion syndrome, pigment granules from the iris pigmented epithelium are deposited on various ocular structures, including the trabecular meshwork. The disorder most frequently affects young myopic individuals. In 1981 Scheie and Cameron documented autosomal dominant inheritance in pigment dispersion syndrome.[88] Specific genes responsible for the human condition have not yet been identified; however, linkage studies suggest that 1 gene is located on chromosome 7q36.[19] Two loci for pigment dispersion syndrome phenotypes similar to human pigmentary glaucoma had been identified in mouse. The first identified locus linked to the pigment dispersion syndrome phenotype, *PDS1*, is located at 7q35-36.[19] A second locus, *PDS2*, was mapped to 18qll-q22.[89] Two genes in the mouse contribute to the disease: *TYRP1* (Tyrosinase related protein 1) and *Gpnmb* (Glycoprotein NMB). Both of these genes are involved in pigment production and/or stabilization of melanosomes.[90] Neither of these genes contribute to the disease in humans.[91] Although, pigment dispersion syndrome is considered to be a secondary glaucoma, identification of the genes causing this condition may have a direct relationship with the pathogenesis of POAG.

## Pseudoexfoliation and Pseudoexfoliative Glaucoma

Pseudoexfoliation syndrome (PEX) is a systemic disease, manifests in a proportion of affected individuals with elevated IOP and glaucomatous damage. Pseudoexfoliation is primarily a disease of the elderly and appears to have wide variation in its prevalence rate in different parts of the world. Despite that, it is rarely visible in a patient before age 55. Some two generation families with pseudoexfoliation have been described.[92] PEX high-pressure glaucomas account for half of all glaucomas in the eastern region of the Arabian peninsula including Oman.[93] A locus on chromosome 2 p16,[94] and *maternal inheritance*, perhaps suggestive of a *mitochondria1 locus*, have been reported.[95]

### 

#### Genetic testing

Genetic testing is now available for humans. It tests 3 of more than 40 mutations for GLC1A, in addition to testing for a mutation of special interest, the MT1 promoter. Whether this mutation is associated with severity or prognosis for glaucoma is controversial and awaits further data.[96]

## Developing a Diagnostic Panel for Patients at Risk for Glaucoma

One of the goals of disease gene discovery is the development of predictive diagnostic tests. For a disease such as glaucoma, where early treatment can be beneficial, diagnostic tests designed to identify individuals at risk for the disease can be particularly valuable. Current testing for glaucoma genes is limited to genes that are known to be associated with glaucoma and is primarily diagnostic, rather than prognostic. Except for specific mutations in the *MYOC* and *OPTN* genes, details regarding the predicted clinical course associated with a glaucoma gene mutation cannot be provided. Genotype-phenotype studies as outlined earlier will help define the prognostic aspects of currently known glaucoma gene mutations. Ultimately the goal is to discover a complete panel of genes that contribute to glaucoma and develop diagnostic and prognostic correlates for the mutations found in each gene. Such a panel would provide a mechanism to identify individuals at risk for the disease and initiate timely treatment before irreversible optic nerve degeneration and blindness occurs.[9]
